# A Perplexing Case of Abdominal Pain That Led to the Diagnosis of Zollinger-Ellison Syndrome

**DOI:** 10.1155/2017/7636952

**Published:** 2017-02-21

**Authors:** Adrienne Lenhart, Mona Hassan, Alireza Meighani, Omar Sadiq, Yousuf Siddiqui

**Affiliations:** ^1^Department of Internal Medicine, Henry Ford Hospital, Detroit, MI 48202, USA; ^2^Division of Gastroenterology and Hepatology, Henry Ford Hospital, Detroit, MI 48202, USA

## Abstract

Zollinger-Ellison syndrome (ZES) is a rare clinical disorder, characterized by hypersecretion of gastric acid and multiple ulcers distal to the duodenal bulb. This occurs via the release of gastrin by neuroendocrine tumors known as gastrinomas. Patients with ZES present with nonspecific GI symptoms, which often leads to a delay in diagnosis. Our patient is a 55-year-old female with chronic abdominal pain, nausea, and diarrhea. She underwent EGD, EUS, MRCP, CT scans, and cholecystectomy, which did not reveal the cause of her symptoms. Repeat EGD showed a cratered ulcer in the second portion of the duodenum, suspicious for ZES. Serum gastrin was initially only moderately elevated while on PPI therapy, but chromogranin A was also elevated. Repeat gastrin level after stopping PPI therapy was 1639 pg/mL. Somatostatin receptor scintigraphy was obtained, which showed two small lesions in the gastrinoma triangle. She subsequently underwent a Whipple pancreaticoduodenectomy and pathology was positive for four microscopic foci of a neuroendocrine tumor. She reported improvement in her symptoms after surgery. This case highlights the need for increased awareness of ZES in patients with unexplained GI complaints and emphasizes the use of multiple modalities in the diagnosis of ZES.

## 1. Introduction

Zollinger-Ellison syndrome (ZES) is a rare clinical disorder, characterized by hypersecretion of gastric acid into the proximal gastrointestinal (GI) tract. The syndrome results from increased secretion of the hormone gastrin by duodenal or pancreatic neuroendocrine tumors, known as gastrinomas [1–4]. The incidence of ZES is rare, ranging from 0.1 to 3 per million in the population each year [1, 5–7]. Patients with gastrinomas are usually diagnosed between the ages of twenty and fifty years old, but cases have been reported in both younger and elderly patients [8]. Around 80% of gastrinomas are sporadic in nature, while approximately 20–30% have been found in association with multiple endocrine neoplasia, type 1 (MEN-1) [9].

The majority of gastrinomas originate in the duodenum, and only around 25% of gastrinomas arise from within the pancreas [8]. However, pancreatic tumors are typically more aggressive and are more likely to metastasize to the lymph nodes, liver, and/or bone than are duodenal tumors [10, 11]. Around 80% of gastrinomas are detected within the gastrinoma triangle, defined as the interface between the confluence of the cystic duct and common bile duct, the junction of the second and third portions of the duodenum, and the junction of the neck and body of the pancreas [12, 13]. Patients with ZES typically present with nonspecific GI symptoms, such as abdominal pain, nausea, vomiting, and chronic diarrhea [2]. Subsequent endoscopic evaluation usually reveals multiple peptic ulcerations distal to the duodenal bulb.

The diagnosis of ZES requires confirmation of hypergastrinemia, and a serum gastrin level over 1000 pg/mL in the setting of gastric pH <2 is virtually diagnostic [1, 8, 14]. Secretin stimulation testing can also be used to differentiate patients with gastrinomas from other causes of hypergastrinemia, such as atrophic gastritis, renal failure, or vagotomy. Once the diagnosis of a gastrinoma has been established, localization and staging of the tumor are sought through endoscopic ultrasound (EUS), contrast enhanced computed tomography (CT), magnetic resonance imaging (MRI), or somatostatin receptor scintigraphy (SRS). We present a rare case of a gastrinoma that highlights the challenges present in making the diagnosis of ZES and illustrates the importance of increased awareness of this syndrome in patients with chronic GI complaints.

## 2. Case

The patient is a 55-year-old Caucasian female with a past medical history of type 2 diabetes, depression, gastroesophageal reflux disease (GERD), and chronic pancreatitis. She had been followed up in the Gastroenterology Clinic for several years, secondary to a history of intermittent, epigastric abdominal pain, nausea, nonbloody emesis, and chronic diarrhea. However, despite extensive testing, a clear etiology of her ailments had not been determined. She initially underwent esophagogastroduodenoscopy (EGD) and EUS that were consistent with erythematous gastropathy and duodenopathy, with pancreatic parenchymal and ductal changes suggestive of chronic pancreatitis. However, no pancreatic masses were visualized on EUS. Magnetic resonance cholangiopancreatography (MRCP) was also unremarkable at that time. Gastric biopsies were consistent with chronic gastritis and staining for* H. pylori* was negative.

**Figure 1:**
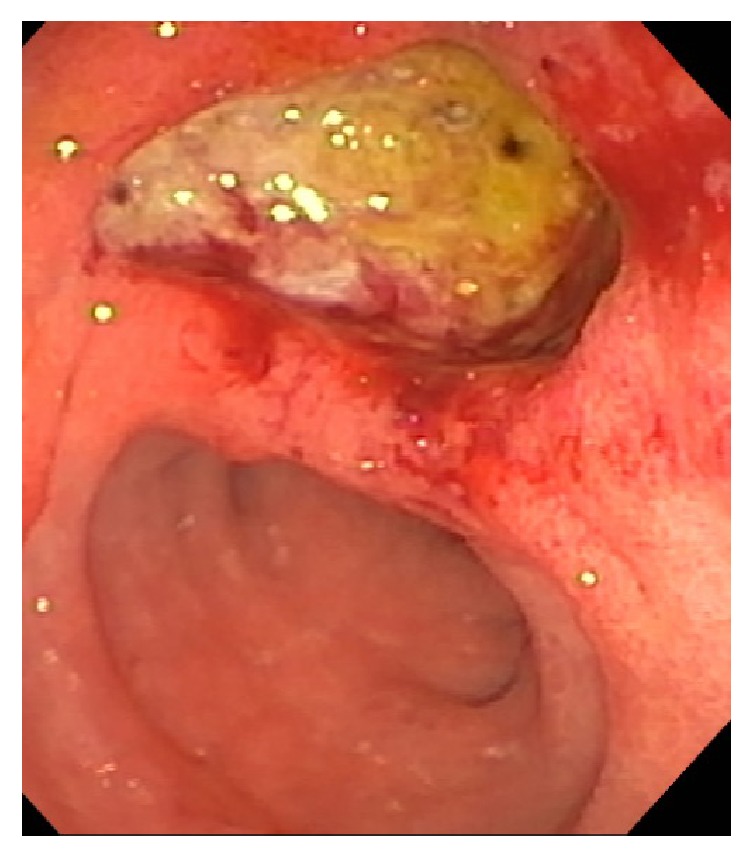
EGD showing 2 cm deeply cratered ulcer in the second portion of the duodenum, suspicious for ZES.

She was hospitalized several times in the interim period with recurrent abdominal pain, presumed to be secondary to GERD and acute on chronic pancreatitis. HIDA scan was consistent with biliary dyskinesia with an ejection fraction of 8% after infusion of cholecystokinin, and she subsequently underwent laparoscopic cholecystectomy. She was started on pancreatic enzyme supplementation in addition to proton pump inhibitor (PPI) therapy, with initial mild improvement in her symptoms. However, she was again hospitalized several months after her cholecystectomy for similar complaints. She underwent repeat EGD, which showed a nonbleeding, deep ulceration at the gastroesophageal junction (LA Grade D), diffuse erythematous mucosa throughout the entire stomach, and a 2 cm deeply cratered ulcer in the second portion of the duodenum (D2) (Figure 1). Gastric and duodenal biopsies were consistent with severe, active chronic gastritis and duodenitis without evidence of malignancy.

**Figure 2:**
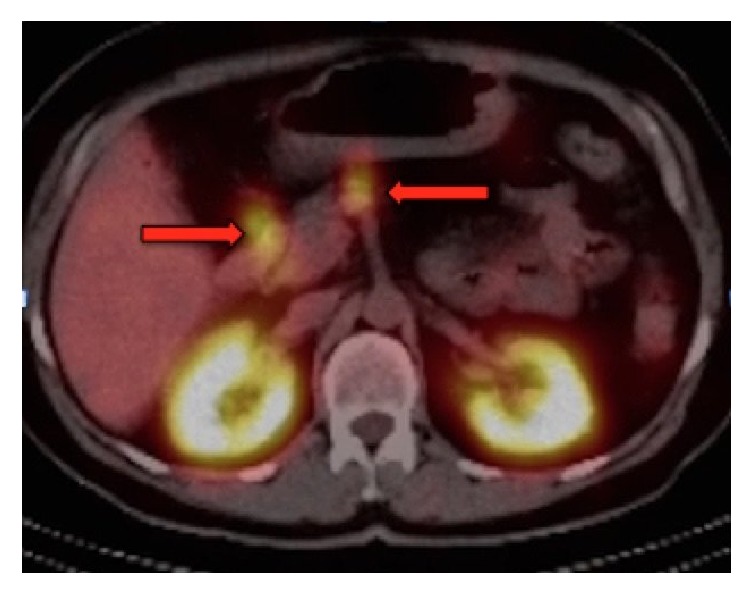
Somatostatin receptor scintigraphy (SRS) showing two small foci of moderately intense abnormal radiotracer uptake within the gastrinoma triangle.

These findings were suspicious for ZES, and appropriate workup was pursued. Serum gastrin level was moderately elevated at 762 pg/mL while on PPI therapy, and chromogranin A was also elevated at 164 ng/mL. Carcinoembryonic antigen (CEA) and carbohydrate antigen 19-9 (CA 19-9) levels were unremarkable. She underwent repeat EUS and CT abdomen/pelvis with contrast, which again did not reveal any pancreatic or duodenal masses. Serum gastrin level was then reevaluated after temporarily discontinuing PPI therapy several months later, and this time it was significantly elevated at 1,639 pg/mL. She subsequently had a SRS scan performed, which revealed two small lesions within the gastrinoma triangle (Figure 2).

**Figure 3:**
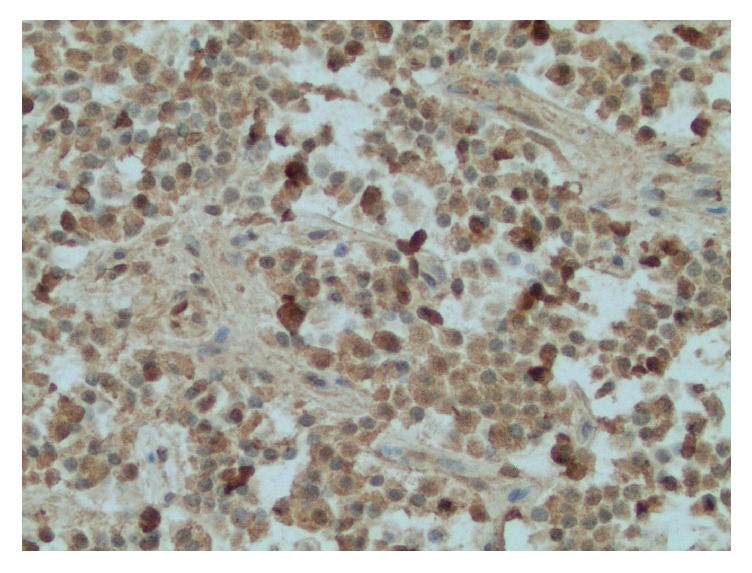
Pathology of pancreatic neuroendocrine tumor with positive staining for gastrin.

She was evaluated by surgical oncology and subsequently underwent a Whipple pancreaticoduodenectomy. Pathology of the pancreas returned positive for four microscopic foci (the largest being 0.1 cm) of a well-differentiated neuroendocrine tumor with metastasis to two peripancreatic lymph nodes (Figure 3). There was also evidence of pancreatic intraepithelial neoplasia, focal chronic pancreatitis, and focal biliary intraepithelial neoplasia. The duodenum and stomach were without significant abnormalities. She is currently maintained on PPI therapy and pancreatic enzyme supplements and has reported improvement in her oral intake and GI symptoms after the surgery.

## 3. Discussion

ZES is characterized by hypersecretion of gastrin and resultant severe peptic ulcer disease (PUD) [1–4]. The rare nature of ZES can lower clinical suspicion for the disorder and ultimately result in a delay in diagnosis, with studies showing the mean time from symptom onset to diagnosis of around 5.9 years [2]. The most common clinical symptoms of nausea, vomiting, and epigastric pain are nonspecific and may lead physicians to first suspect more common disorders such as PUD, GERD, or chronic pancreatitis. However, the symptoms seen in ZES may often be more severe and more refractory to standard medical therapy than are the symptoms seen with PUD caused by nonsteroidal medications or* H. pylori*. This should consequently raise clinicians' suspicion for an alternative diagnosis. In addition, ZES is often characterized by chronic diarrhea (secondary to the high osmotic load of gastric acid as well as malabsorption caused by inactivation of pancreatic enzymes), which would be uncommon with other causes of PUD. These concepts were illustrated in our patient, who was initially diagnosed with GERD and chronic pancreatitis, despite the presence of diarrhea and having recurrent symptoms while on PPI therapy and pancreatic enzyme replacements. If ZES had been entertained earlier in her clinical course, excessive testing may have been avoided, she may have been diagnosed at an earlier stage of disease, and she may have had quicker relief of symptoms.

As previously mentioned, ZES is often suspected when endoscopic evaluation reveals multiple peptic ulcerations or ulcers distal to the first portion of the duodenum in the presence of hypergastrinemia. While gastrin level was elevated in our patient, there were several factors that made the diagnosis of ZES interesting and challenging in this case. For instance, the initial serum gastrin value was less than 1000 pg/mL, which is a nonspecific finding and can be present in other causes of hypergastrinemia other than gastrinomas [8]. In addition, our patient was on PPI therapy at the time of initial testing, which can stimulate a rise in serum gastrin levels secondary to suppression of the normal negative feedback loop to gastrin secretion [15]. It is therefore recommended that patients withhold from PPI therapy for several weeks prior to testing serum gastrin levels [16]. However, despite the only moderately elevated gastrin level while on a PPI, our patient was also noted to have a high serum chromogranin A level, a protein which is elevated in a majority of patients with gastrinomas and often correlates with tumor volume [17]. Chromogranin A levels are not usually elevated in patients with hypergastrinemia secondary to other causes such as chronic atrophic gastritis. Therefore, despite only having a modestly high gastrin level, the elevated chromogranin A level increased clinical suspicion for a gastrinoma in our patient. Serum gastrin was subsequently reevaluated several weeks after discontinuation of her PPI, at which time levels more than doubled.

Once the diagnosis of ZES is established via endoscopy and elevated serum gastrin, imaging should be undertaken to localize the gastrinoma and carry out an evaluation for metastasis. CT and MRI are both highly accurate in detecting lesions greater than 3 cm; however, accuracy decreases for tumors that are smaller than 2 cm in size (studies have reported around 42–56% sensitivity of CT scans and 25–83% sensitivity for MRI) [18, 19]. Gastrinomas express receptors for somatostatin, allowing SRS scans to be utilized for tumor detection with a higher sensitivity than CT/MRI (reported to be around 80–85%) [13, 19]. Recently, EUS has also become an important imaging study and allows for the identification of pancreatic tumors as small as 2–5 mm in diameter, with sensitivity reaching up to 87% [19–21]. Our patient underwent extensive imaging with CT, MRI, and EUS before the tumor was finally identified using SRS. While the reasons why her gastrinoma was not initially detected are not completely known, a large component may be secondary to the very small size of the tumors, as the largest measured only 0.1 cm. This emphasizes that the use of multiple imaging modalities, including SRS, may be imperative in the diagnosis of a gastrinoma.

## 4. Conclusion

In conclusion, this case provides a unique example of ZES that highlights the importance of increased awareness of this disorder in patients who present with chronic GI symptoms. This case also supports the use of chromogranin A to either increase or decrease clinical suspicion for a gastrinoma when serum gastrin levels are only moderately elevated. In addition, this case emphasizes the use of multiple endoscopic and imaging modalities in the ultimate diagnosis of ZES.
